# Enhanced rehabilitation after surgery: principles in the treatment of emergency complicated colorectal cancers – a narrative review

**DOI:** 10.25122/jml-2025-0049

**Published:** 2025-03

**Authors:** Alexandra-Ana Mihailescu, Sebastian Gradinaru, Alin Kraft, Corneliu-Dan Blendea, Bogdan-Sorin Capitanu, Stefan Ilie Neagu

**Affiliations:** 1 Faculty of Medicine, Carol Davila University of Medicine and Pharmacy, Bucharest, Romania; 2 Department of Anesthesiology and Critical Care, Foisor Clinical Hospital of Orthopedics, Traumatology, and Osteoarticular Tuberculosis, Bucharest, Romania; 3 Faculty of Medicine, Titu Maiorescu University, Bucharest, Romania; 4 Department of General Surgery, Ilfov County Emergency Clinical Hospital, Bucharest, Romania; 5 Department of General Surgery, General Doctor Aviator Victor Atanasiu National Aviation and Space Medicine Institute, Bucharest, Romania; 6 Department of Medical-Surgical and Prophylactic Disciplines, Faculty of Medicine, Titu Maiorescu University, Bucharest, Romania; 7 Department of Recovery, Physical Medicine and Balneology, Ilfov County Emergency Clinical Hospital, Bucharest, Romania; 8 Department of Orthopedics, Foisor Clinical Hospital of Orthopedics, Traumatology and Osteoarticular Tuberculosis, Bucharest, Romania

**Keywords:** emergency colorectal surgery, complicated colorectal cancer, perioperative care, multimodal rehabilitation, APACHE II, Acute Physiology and Chronic Health Evaluation, ASA, American Society of Anesthesiologists, ELPQuiC, Emergency Laparotomy Pathway Quality Improvement Care, ERAS, Enhanced Recovery After Surgery, GDFT, Goal-Directed Fluid Therapy, MAP, Mean Arterial Pressure, NGT, Nasogastric Tube, PECS, Pectoral Nerve Block, PONV, Postoperative Nausea and Vomiting, POSSUM, Physiological and Operative Severity Score for the Enumeration of Mortality, P-POSSUM, Portsmouth-POSSUM, SIRS, Systemic Inflammatory Response Syndrome, SSR, Surgical Stress Response, TAP, Transversus Abdominis Plane

## Abstract

Enhanced Recovery After Surgery (ERAS) protocols are used in elective colorectal surgeries and have shown improved recovery for many patients. However, using these protocols in emergency colorectal surgery, especially in complicated cases of obstructive colorectal cancer, is still debated. This review examined the ERAS principles that can be adapted for emergencies. We reviewed the literature on applying ERAS principles in emergency colorectal cancer surgery. We analyzed key strategies used before, during, and after surgery. The aim of ERAS in emergency colorectal surgery is to reduce physical stress from urgent surgical conditions. Before surgery, the focus should be on early patient recovery, managing blood sugar levels, and providing patient education when possible. Minimally invasive techniques, careful fluid management, and effective pain relief during surgery are intraoperative key points. After surgery, early feeding, patient mobilization, and minimizing the use of medical devices are encouraged. Studies have shown that using ERAS in emergencies can lower mortality, reduce hospital stays, and influence patient recovery rates, although it may lead to higher initial costs. Still, following ERAS in emergencies is inconsistent due to logistical issues and patient health changes. More people are starting to recognize the benefits of ERAS in obstructive colorectal cancer surgery. Although there is less evidence compared to elective procedures, new studies suggest that organized steps for care can improve patient outcomes. Further research is needed to improve ERAS emergency protocols and identify patients suitable for this approach so that healthcare resources can be used better.

## INTRODUCTION

Enhanced Recovery After Surgery (ERAS) is a protocol to reduce surgical stress and support organ recovery. For patients undergoing emergency laparotomy, physiological issues such as bowel dysfunction, insulin resistance, fluid shifts, and systemic inflammatory response syndrome (SIRS) can arise even before surgery [1]. Hypovolemia in emergency surgical patients can severely affect kidney function and circulation, so resuscitation should accompany diagnostic and surgical preparation.

ERAS is a commonly adopted approach for perioperative management in elective colorectal procedures, contributing to reduced postoperative complications, shorter hospital stays, and enhanced recovery. Furthermore, it is also cost-efficient. Recent research indicates that ERAS principles can also be successfully adapted for urgent colorectal cases, such as acute obstruction caused by a tumor [2].

This narrative review examines the principles of ERAS and their application in managing obstructed colorectal cancers. By assessing current evidence, this study aimed to address essential perioperative strategies and interventions tailored to patients that accelerate recovery and improve outcomes for this high-risk surgical population.

## HISTORY OF THE ERAS CONCEPT

ERAS has transformed surgical care since its inception in the 1990s, initially focusing on elective colorectal procedures. Pioneered by Professors Henrik Kehlet from the Center for Surgical Pathophysiology and Douglas W. Wilmore from Boston University, ERAS establishes new benchmarks for patient recovery and surgical outcomes [3,4].

Fast-track surgery has evolved due to advances in evidence-based postoperative care [5]. Studies examining the effects of standard or conventional care have demonstrated that many traditional approaches, such as preoperative bowel preparation, nasogastric tubes and intraoperatively placed drains, enforced bed rest, and gradual diet progression, are unnecessary or potentially harmful [6,7].

Some authors suggest that the combined influence of individual strategies contributes to improved recovery [8,9]. This idea is further sustained by a study that compared modified versions of an ERAS program, where one group of patients underwent laparoscopic surgery while the other underwent open surgery. Although better outcomes were anticipated in the laparoscopic group, the findings indicated that both groups had comparable results [10].

In 2011, Møller *et al*. evaluated mortality rates among patients with perforated peptic ulcers within a multimodal, multidisciplinary perioperative care program. Their findings showed that adherence to this approach led to a more than one-third reduction in the 30-day mortality rate compared to conventional treatment [11].

A 2014 UK study assessed a structured care program for optimizing emergency laparotomy pathways (ELPQuiC) and found a significant reduction in mortality. This ERAS-based protocol focused on early assessment using warning scores, prompt antibiotic administration, surgery within 6 hours of decision-making, individualized hemodynamic management, and enhanced postoperative care. An economic analysis revealed higher hospital costs but reduced mortality and overall societal expenses [12].

Similarly, in 2017, Tengberg *et al*. evaluated the outcomes of a protocol that involved ERAS principles in 600 high-risk patients undergoing acute abdominal surgery. The study concluded that patients who followed the ERAS principles had lower mortality rates than cohorts receiving conventional care [13].

## CURRENT CONCEPTS IN ERAS

To our knowledge, few comparative studies compare cohorts of patients that followed conventional versus ERAS protocols-based care for colorectal cancers requiring emergency operations.

The first study, conducted by Lohsiriwat *et al*., compared two groups of patients who underwent surgery for obstructive colorectal cancer. The first group included 40 patients who received conventional postoperative care, while the second group included 20 patients who followed an ERAS protocol. The results favored the ERAS approach, showing better outcomes than standard care. Patients in the ERAS group experienced shorter hospital stays, faster gastrointestinal recovery, and earlier initiation of adjuvant therapy for the underlying condition [2].

Wisely *et al*. conducted a retrospective study comparing two patient groups: one managed using ERAS protocols and the other treated without these principles. The study included patients who underwent emergency abdominal surgery for conditions such as obstruction (the most common), inflammatory diseases, and ischemia. The findings showed a significant reduction in major complications in patients managed with ERAS principles (*P* = 0.002). However, no differences were observed in mortality rates, hospital readmissions, or length of hospital stay [14].

Another study assessing the effectiveness of ERAS protocols in obstructive colorectal cancer was conducted by Shida *et al*. This study compared two patient groups: one followed the ERAS protocol, and the other received conventional care. Consistent with previous findings, the results favored the ERAS approach. The hospitalization duration was significantly reduced in the ERAS group compared to those receiving standard care (*P* < 0.05), indicating a faster recovery without an increased risk of complications, readmission, or reintervention. Mortality rates remained similar between the two groups [15].

Shang *et al*. conducted a study on a larger cohort of 839 patients who underwent surgery for obstructive colorectal cancer. The primary objective was to compare the outcomes of patients managed with ERAS protocols to those receiving traditional postoperative care. Gastrointestinal function recovery was assessed based on the return of defecation or gas passage, with results showing a faster recovery in the ERAS group, leading to a shorter hospital stay. Additionally, postoperative complications were significantly lower in the ERAS group (*P* = 0.002). However, no differences were observed in surgery-related complications, mortality, or readmission rates [16].

The last study, conducted by Mihailescu *et al*., followed a similar methodology, comparing two groups of patients who underwent surgery for obstructive colorectal cancer. One group adhered to the ERAS protocol, and the other followed a conventional postoperative care program. The findings again favored ERAS, showing a shorter hospital stay and lower complication rate, although this did not reach statistical significance. Additionally, readmission rates were similar between the two groups, while the reoperation rate was higher in the traditional care group [17].

The guidelines recommended by the ERAS Society for perioperative care in elective settings include 20–21 ERAS principles for colorectal surgery [18-20]. Most of these principles have been adapted for emergency colorectal surgery with some modifications:

### Preoperative principles

- Patient education and counseling
- Medical optimization
- Glycemic control

### Intraoperative principles

- Use of epidural anesthesia/analgesia
- Goal-directed fluid therapy (GDFT)
- Prevention of hypothermia
- Prophylaxis for postoperative nausea and vomiting (PONV)
- Preference for minimally invasive surgical techniques
- Avoidance of intraperitoneal drainage tubes

### Postoperative principles

- Multimodal analgesia
- Early removal of the nasogastric tube
- Early resumption of oral feeding
- Early removal of urinary, peripheral, and central venous catheters
- Respiratory physiotherapy
- Early active postoperative mobilization

Using these ERAS principles, surgical teams can improve patient outcomes, reduce hospital stays, and accelerate recovery following colorectal surgery.

## PREOPERATIVE PRINCIPLES

### Education and counseling

When it comes to elective surgeries, taking the time to educate and counsel patients and their families can make a big difference in easing stress, pain, and anxiety after the procedure. While substantial evidence supports these benefits in elective cases, data on emergency colorectal surgery remains limited. In urgent situations where postoperative complications are a significant concern, healthcare providers must educate patients and their families before surgery. This includes explaining the rationale behind the surgical decision, the type of procedure being performed, potential intraoperative and postoperative risks, the possibility of requiring a stoma, and the expected hospital stay. This information helps patients and their families feel more informed, prepared and reassured [21].

For high-risk patients, it is essential to discuss care protocols before proceeding with surgery, even if doing so can be difficult in urgent situations. Objective mortality scores can aid these discussions and should be utilized with other evaluations, like frailty scores [22]. Shared decision-making and personalized care can be challenging for patients with surgical risks in discussions with senior physicians like surgeons and anesthesiologists. While validated risk scores can guide these conversations, it is important to recognize their limitations for individual patients. Establishing treatment escalation plans and managing pain and acute physiological disturbances related to abdominal conditions is also essential [23].

Patients and their families should discuss and document care plans in advance. A stoma can lead to longer hospital stays, especially without proper preoperative education. However, education from a specialized stoma care nurse can reduce hospital stays, improve quality of life, and enhance psychosocial adjustment for stoma patients [24].

### Medical optimization

Preoperative optimization involves two key components: enhancing patient conditions and improving healthcare system resources, including facility access [25]. In surgical emergencies, time limitations may restrict thorough evaluation and optimization. Therefore, risk stratification is essential in the standard preoperative assessment for emergency colorectal surgery [26].

The American Society of Anesthesiologists (ASA) classification is frequently used to assess risk, but other scoring systems may better predict the risk of death after surgery. For patients undergoing emergency surgery, several scoring systems are commonly used, including the Acute Physiology and Chronic Health Evaluation (APACHE II), the Physiological and Operative Severity Score for the Enumeration of Mortality (POSSUM), its modified version Portsmouth-POSSUM (P-POSSUM), and the Modified Early Warning Score, which help assess patient risk and predict outcomes. It is important to note that when early warning scores worsen, they are strongly linked to a higher risk of death than stable or improved scores [27].

Patients with an estimated mortality risk exceeding 10% who require emergency laparotomy should be admitted to the intensive care unit for postoperative management and potentially for preoperative care to optimize their overall condition [28]. It is important to highlight that individuals receiving goal-directed optimization (with a central venous pressure of 8–12 cmH_2_O, a mean arterial pressure of at least 65 mmHg, and a urine output of no less than 0.5 mL/kg per hour) have shown lower mortality rates and shorter hospital stays in various studies [29,30]. These findings suggest goal-directed preoperative optimization, such as intravenous fluid stabilization and antibiotic administration, could enhance outcomes following major emergency abdominal surgery.

### Glycemic control

Hyperglycemia increases the risk of surgical complications in both diabetic and non-diabetic patients. It is essential to consider long-term glycemic control and blood glucose levels during presentation. High blood sugar can impair neutrophil function and cause excessive production of reactive oxygen species and inflammatory mediators, leading to cellular damage and dysfunction in the vascular and immune systems [31]. Hyperglycemia results from the physiological stress response, which contributes to the development of insulin resistance and serves as an indirect indicator of tissue damage. Implementing insulin therapy to manage hyperglycemia can mitigate associated complications [32,33].

High hemoglobin A1c (HbA1c) levels before surgery and elevated blood sugar during surgery can lead to serious complications, especially in emergency colorectal procedures. Although optimizing HbA1c can be difficult, controlling blood sugar is essential for all patients. Doctors should consider preoperative blood sugar levels, the type of surgery, and the patient’s overall health in their management [34,35].

## INTRAOPERATIVE PRINCIPLES

### Epidural anesthesia/analgesia

In ERAS protocol, thoracic epidural analgesia may improve pain control and expedite bowel recovery. However, evidence does not show it leads to better overall recovery or fewer complications compared to other methods like patient-controlled opioids or continuous wound infiltration [36].

More studies are needed to examine different pain relief methods that fit into the ERAS program. Pain management is a key point of ERAS protocols, enabling better recovery and adherence to rehabilitation programs [37]. Different techniques of regional anesthesia, such as transversus abdominis plane (TAP) block, rectus sheath block, or pectoral nerve block (PECS), are alternatives that offer significant benefits. They may allow for quicker recovery than epidural anesthesia, which is being reconsidered due to concerns about its risks compared to newer pain management methods.

No study has explored explicitly whether these techniques protect the surgical stress response (SSR) as an alternative to epidural anesthesia. Nevertheless, they do offer the benefit of reducing sympathetic stimulation and opioid consumption. The potential protective effect of epidural anesthesia may be restricted with regional blocks, as most new techniques deliver only parietal analgesia, lacking visceral support.

Epidural anesthesia has been shown to reduce cortisol and epinephrine levels while increasing insulin levels, and it may also enhance immune response modulation [38-40]. ERAS protocols tailored for cancer patients, which prioritize the use of epidural anesthesia, may differ significantly from those for non-cancer patients, where less invasive techniques are often preferred. Additionally, some research indicates that regional anesthesia may decrease the likelihood of cancer recurrence [41].

### Goal-directed fluid therapy (GDFT)

Goal-directed fluid therapy (GDFT) optimizes cardiac index in high-risk surgical patients. Maintaining a mean arterial pressure (MAP) of 60–65 mmHg and the cardiac index at 2.2 L/min/m^2^ using vasopressors and inotropes is essential. Careful monitoring of fluid balance is crucial, with intraoperative fluid therapy adjusted based on assessments of hypovolemia [42]. Despite challenges in achieving a zero fluid balance during emergencies, especially with factors like fasting and hemorrhage, it remains a crucial goal for effective patient care. GDFT is crucial for maintaining organ perfusion and ensuring effective oxygen delivery during and after surgery. Advanced devices, like transesophageal Doppler monitors, are often required for GDFT implementation. A recent meta-analysis confirmed that GDFT within an ERAS protocol significantly reduces the duration of intensive care and the time to the first bowel movement, although it does not influence mortality rates, time to the first passage of gas, or the risk of postoperative ileus [43].

### Prevention of hypothermia

Patients are susceptible to hypothermia due to multiple factors. One of them is exposure to cold environments in the operating room. Another factor is the effect of anesthesia and the administration of cold intravenous fluids. Hypothermia influences drug metabolism and coagulation factors and could increase the risks of bleeding or wound and cardiac complications as well. To prevent hypothermia, the use of warming mattresses or forced-air warming blankets is advisable. Additionally, fluid warmers should be employed to ensure that intravenous fluids and blood products are administered at an appropriate temperature [44].

Intraoperative and postoperative hypothermia can affect up to 60% of patients undergoing emergency surgeries, particularly those facing major abdominal procedures or receiving large volumes of intravenous crystalloids. Although no significant correlation has been established between hypothermia and the length of hospital stays in critically ill patients, it may contribute to delayed recovery and elevate the risk of postoperative complications, including infections, major cardiac events, and excessive blood loss [45,46].

### Prophylaxis for nausea and vomiting (PONV)

After emergency laparotomy, patients often face a significant risk of nausea and vomiting due to a range of physiological changes and gastrointestinal injuries. To enhance patient comfort, it is essential to minimize the use of intravenous opioids by adopting a multimodal approach [47]. Various effective antiemetic options are available, including serotonin or dopamine antagonists, antihistamines, and corticosteroids, which can greatly improve outcomes.

Dexamethasone is the most commonly used drug for prophylaxis of PONV due to its accessibility and effectiveness. Its action targets both central and peripheral pathways by inhibiting prostaglandins, generating anti-inflammatory agents, and decreasing the production of endogenous opioids [48]. Nonetheless, a recent review indicated that administering 4–5 mg of dexamethasone for PONV produces effects similar to 8–10 mg, whether used alone or alongside other antiemetic medications [49].

Concerns have been raised regarding wound healing and the immunosuppressive effects of dexamethasone in patients undergoing surgery for colorectal cancer. Due to the limited number of studies on this topic, definitive conclusions cannot be drawn. Nevertheless, given its potential influence on cancer progression, caution is advised when using dexamethasone for the PONV in patients with cancer. Furthermore, corticosteroids, more so than non-steroidal anti-inflammatory drugs (NSAIDs), may negatively influence anastomotic healing, thereby increasing the risk of postoperative fistulas and wound infections [50].

### Minimally invasive surgery

A fundamental aspect of ERAS strategies is the implementation of minimally invasive surgery whenever possible. Laparoscopy lowers stress responses, reduces complications, decreases hospital stays, and lowers mortality rates. It is now considered the gold standard for numerous surgical procedures. Furthermore, it minimizes neurohumoral activation, enhancing recovery by reducing wound size and releasing postoperative inflammatory factors [51,52].

Following ERAS protocols and utilizing laparoscopic surgery are particularly important for patients with cancer. As mentioned, laparoscopic surgery reduces stress response, lowering pro-inflammatory cytokines and C-reactive protein levels [53]. This reduction may decrease immune impairment and enhance the body's immune defense against cancer. Additionally, patients who undergo laparoscopic surgery often begin adjuvant chemotherapy sooner after colorectal cancer surgery [54].

A recent systematic review and meta-analysis comparing emergency laparoscopic and open colorectal surgery found that laparoscopic surgery, involving 7,865 procedures versus 55,862 open surgeries, had lower mortality, reduced overall morbidity, fewer wound infections, wound dehiscence, ileus, and cardiac and pulmonary complications, along with shorter hospital stays [55]. However, most studies were non-randomized retrospective cohorts, raising concerns about selection bias.

In conclusion, laparoscopic surgery is a valuable option that should be contemplated when circumstances permit. The decision to employ this minimally invasive technique hinges on several critical factors, including the specific underlying medical conditions being addressed, the availability and adequacy of surgical resources such as equipment and skilled personnel, and the level of expertise possessed by the surgeon. Each element is pivotal in determining whether laparoscopic surgery is the most suitable method for a patient’s treatment plan.

### Intraperitoneal drainage tubes

Intraperitoneal drain tubes are commonly used to prevent and remove fluid accumulation in the peritoneal cavity. However, their effectiveness as a preventive measure following major elective abdominal surgery has not been established. In patients undergoing elective surgery, the evidence supporting any potential benefits of using these drain tubes is weak or nonexistent [56]. Research indicates that patients with intra-abdominal drains experience similar rates of complications and mortality compared to those who do not have drains. A meta-analysis of four randomized controlled trials involving patients undergoing rectal surgery found no advantages to using closed drainage systems [57]. A recent prospective study involving 1,805 patients who underwent elective colorectal surgery revealed that drains did not result in lower rates or earlier detection of collections. Instead, the study found that drains were associated with delayed hospital discharge and an increased risk of surgical site infections [58].

The need for peritoneal drainage following emergency laparotomy remains a subject of debate within the medical community. Current evidence does not support the routine use of drainage after emergency colorectal resections [59]. Numerous authors advocate for the avoidance of intra-abdominal or pelvic drainage except in specific circumstances, such as significant intraoperative hemorrhage, the presence of purulent or fecal peritonitis, and fragile anastomoses [60]. In summary, prophylactic usage of intra-abdominal drains is not recommended due to insufficient evidence demonstrating their efficacy in clean and low-contaminated surgical cases. This practice may be influenced in cases of contaminated abdominal surgery.

## POSTOPERATIVE PRINCIPLES

### Multimodal analgesia

Postoperative pain management is essential, but guidelines adherence in emergency surgeries might be challenging. Experts recommend personalized multimodal analgesia that combines non-pharmacological and pharmacological methods tailored to the patient and the surgery type [61]. Pain directly activates the hypothalamic-pituitary axis and the sympathetic nervous system, leading to immunosuppression [62]. While no definitive evidence indicates that opioids worsen cancer outcomes, it is crucial to implement opioid-sparing strategies as integral components of ERAS protocols [63]. These strategies are essential for minimizing opioid consumption and maintaining immune homeostasis, with regional anesthesia and multimodal analgesia serving as vital tools in achieving these goals [64].

By minimizing postoperative pain and reducing the need for systemic opioids, epidural anesthesia contributes to improved patient outcomes, increased mobility, and a faster return to normal activities following surgery. Its role in ERAS protocols underscores the importance of pain management strategies prioritizing comfort and recovery efficiency.

Ultimately, ongoing pain after surgery can hinder cancer progression, as it may necessitate prolonged opioid use and impede access to critical therapies such as radiotherapy and chemotherapy. Essential components of ERAS protocols are integral to 'protective anesthesia', which is a novel anesthetic approach that utilizes completely opioid-free pain management methods (such as a total neuraxial block) to minimize hyperalgesia and avert central sensitization and lasting postoperative pain [65].

Meta-analyses indicate that intravenous (IV) lidocaine infusions in elective abdominal surgery patients can reduce postoperative pain, lower opioid consumption, and shorten hospital stays, likely by facilitating earlier recovery of gastrointestinal function [66]. Furthermore, sub-anesthetic ketamine infusions (0.1–0.5 mg/kg/h) have become increasingly popular for postoperative analgesia, helping to decrease opioid requirements. However, higher doses of ketamine may elevate the risk of adverse effects, such as hallucinations and delirium, which may necessitate dose adjustments [67].

### Prompt removal of the nasogastric tube (NGT)

The evidence surrounding gastric decompression remains unclear, and a selective approach to postoperative nasogastric tube (NGT) use is more effective than routine application, even among emergency surgery patients. Following surgery, when gastric aspirate volumes are adequately managed, the NGT should be removed to promote oral intake.

Prophylactic NGT placement after abdominal surgery aims to reduce complications such as nausea and gastric distension. However, evidence suggests that NGT insertion may be unnecessary in emergency laparotomy cases. A study by Sapkota *et al*. conducted a prospective controlled trial on 115 patients to assess the need for prophylactic decompression using NGT after emergency laparotomy. The patients were divided into two groups: one with NGT placement and one without. The results showed no significant differences in complications or the return time of gastrointestinal function [68].

Nevertheless, certain patients still require NGT placement. If adherence to ERAS protocols is pursued, prompt removal is recommended. The main concern is whether early removal leads to an increased reinsertion rate. A study by Venara *et al*. found that immediate NGT removal did not increase the need for reinsertion. Instead, factors such as left-sided colon cancer, postoperative ileus, and severe postoperative complications were identified as influencing the likelihood of NGT reinsertion [69]. In conclusion, the decision to use an NGT should be made individually, considering the potential risks of gastric stasis and aspiration associated with intestinal dysfunction. The necessity of the NGT should be reassessed daily, and it should be removed as soon as it is no longer required. The NGT is indicated for patients experiencing ileus or considerable intestinal edema following surgery.

### Early restart of oral feeding

Managing nutrition after an emergency laparotomy is crucial, even if preoperative optimization is not possible. Early feeding is beneficial, even in emergencies. If stomach feeding is not possible, early parenteral nutrition can help reduce the time without proper nutrition. Once patients' condition improves and restrictions are lifted, they can transition back to oral or enteral nutrition when safe.

Klappenbach *et al*. conducted a study that included 295 patients who underwent emergency laparotomy, comparing early feeding (as per ERAS protocols) with a liquid diet initiated after bowel function returned (following traditional care protocols). The study found no significant differences in morbidity, postoperative ileus, or hospital stay between the two groups, but the early-feeding group had a higher rate of vomiting [70]. Early feeding led to shorter hospital stays in a separate retrospective study of 84 patients who had emergency bowel resection [71]. While early intake after colorectal surgery is feasible, more well-designed trials are required for conclusive evidence. Feeding should be adjusted based on any signs of postoperative ileus, and the causes of feeding intolerance must be investigated and addressed.

### Prompt removal of catheters

Urinary catheters are frequently used in patients with major abdominal surgery to relieve bladder pressure, track urine output, and avoid urinary retention. ERAS protocols for elective surgical procedures advocate for the prompt removal of catheters in the postoperative period to promote patient mobility, improve comfort, and decrease the risk of urinary tract infections, which are more likely with extended catheter use [72,73]. A study by Meillat *et al*. evaluated the feasibility of early urinary catheter removal following rectal cancer surgery. Their findings suggested that removal on the third postoperative day was feasible in approximately 90% of patients and was not linked to complications [74]. In older patients, using a urinary catheter greatly increases the risk of postoperative delirium. This is due to infection, discomfort, and limited mobility [75-77]. Bo *et al*. conducted a study on 1,867 hospitalized patients and found that urinary catheter use was associated with an increased risk of delirium and cognitive impairment [78]. However, in urgent surgical circumstances, it may be essential to closely observe urine output and fluid balance for individuals experiencing sepsis or significant physiological disturbances [66]. While surgical techniques can achieve source control, ongoing resuscitation and urinary catheterization may be necessary beyond the first postoperative day following an emergency laparotomy. Additionally, catheters may be required for genitourinary procedures, postoperative immobility, or during sedation [79,80]. Except for these circumstances, when rigorous fluid management is no longer necessary, the urinary catheter should be removed immediately, and movement should be promoted.

### Respiratory physiotherapy

A recent randomized controlled trial involving 150 patients who underwent exploratory laparotomy, including around 45% undergoing emergency colorectal surgery, indicated that adding incentive spirometry to standard breathing exercises did not significantly enhance postoperative lung function, reduce respiratory complications, or decrease the length of hospital stays [81]. A 2012 systematic review and meta-analysis found that postoperative deep breathing exercises improved lung function and respiratory muscle strength after elective upper abdominal surgery but did not reduce pulmonary complications [82]. While no direct evidence shows the benefits of breathing exercises after emergency colorectal surgery, many surgeons still recommend that their patients participate in early postoperative deep breathing and coughing exercises. These exercises typically include supervised training for sputum clearance, strengthening of the inspiratory muscles, and deep breathing techniques.

### Early active mobilization after surgery

Staying in bed for a long time increases the risk of lung problems, blood clots, insulin resistance, and weak muscles. Getting up and moving early is especially important for patients in emergencies. This includes older people who may already have weak muscles and those with sepsis, who are at a higher risk of losing muscle. Following emergency surgery, elderly patients are at a major risk of losing their ability to function independently. Early movement after surgery may not have clear benefits, but staying in bed too long can lead to pneumonia, blood clots, insulin resistance, and muscle weakness [83]. ERAS guidelines mandate early mobilization (either assisted or independent) following elective colorectal surgery. This practice is critical for optimal recovery [84]. Despite these recommendations, early mobilization or physical therapy after emergency colorectal surgery remains underexplored. An Australian multicenter trial is currently evaluating a physiotherapy program that incorporates breathing exercises and physical activity to reduce complications and facilitate recovery after emergency laparotomy [85]. Additionally, another study has demonstrated that early mobilization in the ICU after surgery leads to shorter hospital stays and enhances functional capacity at discharge [17].

## ECONOMIC IMPLICATIONS AND FUTURE DIRECTIONS

Implementing ERAS protocols in emergency colorectal and other cancer surgeries is a cost-effective approach that can help healthcare systems. Research shows that ERAS protocols shorten hospital stays, reducing complications and overall costs [86]. For example, a study in Alberta, Canada, by Thanh *et al*. found that using ERAS principles in colorectal surgeries saved about $1,768 per patient, mainly due to fewer days in the hospital and fewer postoperative issues [87]. By applying ERAS protocols, hospitals can better manage their resources and improve patient care.

Future improvements may focus on adapting protocols specifically for emergency surgeries and expanding their use for different types of cancer. Ongoing research aims to make ERAS even more effective in reducing complications, speeding up recovery, and lowering costs. Widespread use of ERAS could also lead to a more standardized approach to surgical care, improving patient outcomes and making healthcare systems more efficient [88].

## CONCLUSION

This review highlights the growing importance of ERAS in emergency colorectal surgery, with more research expected to refine its application. Studies consistently show that ERAS protocols can enhance recovery while maintaining patient safety. The advancement of 'fast-track' surgery relies on three key aspects: identifying effective strategies to improve recovery, integrating these approaches into structured treatment plans, and ensuring hospitals adhere to ERAS guidelines to optimize patient outcomes.
